# A Review on Flavonoid Apigenin: Dietary Intake, ADME, Antimicrobial Effects, and Interactions with Human Gut Microbiota

**DOI:** 10.1155/2019/7010467

**Published:** 2019-10-16

**Authors:** Minqian Wang, Jenni Firrman, LinShu Liu, Kit Yam

**Affiliations:** ^1^Food Science Department, Rutgers University, New Brunswick 08901, USA; ^2^Dairy and Functional Food Research Unit, Eastern Regional Research Center, ARS, USDA, Wyndmoor 19038, USA

## Abstract

Apigenin is a flavonoid of low toxicity and multiple beneficial bioactivities. Published reviews all focused on the findings using eukaryotic cells, animal models, or epidemiological studies covering the pharmacokinetics, cancer chemoprevention, and drug interactions of apigenin; however, no review is available on the antimicrobial effects of apigenin. Research proves that dietary apigenin passes through the upper gastrointestinal tract and reaches the colon after consumption. For that reason, it is worthwhile to study the potential interactions between apigenin and human gut microbiota. This review summarizes studies on antimicrobial effects of apigenin as well as what has been reported on apigenin and human gut microbiota. Various levels of effectiveness have been reported on apigenin's antibacterial, antifungal, and antiparasitic capability. It has been shown that apigenin or its glycosides are degraded into smaller metabolites by certain gut bacteria which can regulate the human body after absorption. How apigenin contributes to the structural and functional changes in human gut microbiota as well as the bioactivities of apigenin bacterial metabolites are worth further investigation.

## 1. Introduction

**Figure 1:**
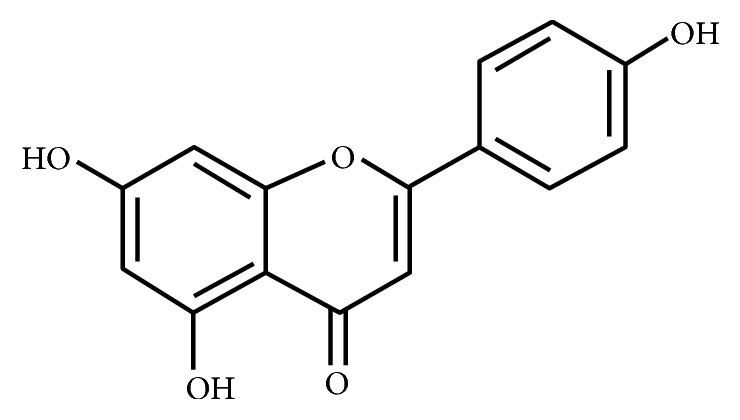
Apigenin (4′,5,7-trihydroxy-flavone).

Flavonoids are a type of phytochemicals called polyphenols, which are the secondary metabolites produced by plants [1]. By producing flavonoids, plants possess a defense mechanism to protect against UV-B and ward off microbial infection and herbivory [2]. “The Handbook of Natural Flavonoids” published in 1999 contains information on 6467 known flavonoid structures with formulae, references, and information on biological activities [3]. Among the over 6000 different flavonoids, quercetin, kaempferol, myricetin, apigenin, and luteolin are the five most ubiquitous plant flavonoids [4]. Apigenin, 4′,5,7-trihydroxy-flavone (Figure 1), is one of the predominant monomeric flavonoids found in a daily diet [5]. Based on the chemical structure of its backbone, apigenin is a flavone, one of the subclasses of flavonoids. Apigenin has gained attention among researchers partly due to its low toxicity and multiple beneficial bioactivities.

The largest number of published reviews on apigenin focus on its effects on various cancers [4, 6–14]. Those reviews include findings relating to the pharmacokinetics, cancer chemo-prevention, and drug interactions of apigenin based on eukaryotic cells, animal models, or epidemiological studies. However, no review on the antimicrobial effects of apigenin exists, although many research papers have reported on the bacterial inhibitory activities from natural flavonoids. Another interesting aspect of the effects of apigenin on bacteria is its interactions with gut microbes. Apigenin has a low solubility [15, 16] and a low bioavailability [4], and thus it may come into contact with the colon microbiota and be metabolized into smaller and more bioavailable molecules [17]. Apigenin may also affect the composition and functionality of gut microbiota. Therefore, there may exist interactions between them. The objective of this present review is to fill the knowledge gap by providing an overview of reported results from antimicrobial tests using apigenin. To that end, the dietary sources of apigenin, certain relevant chemical and biological properties, ADME (absorption, distribution, metabolism, and excretion) of apigenin, the antimicrobial effects of apigenin and its interactions with gut microbiota will be presented and discussed in this review.

## 2. Apigenin: Dietary Source and Daily Intake Levels

Before the discussion of the effects of dietary apigenin on gut bacteria, it is necessary to look at the dietary intake level of apigenin. The distribution of apigenin in the plant kingdom is wide, as it has been found in many vegetables, herbs, and fruits [5]. Fresh parsley, vine spinach, celery seed, green celery heart, Chinese celery, and dried oregano are dietary sources with high apigenin content [5]. Other plants in which apigenin has been identified include red and white sorghum, rutabagas, oranges, kumquats, onions, wheat sprouts, tea, and cilantro [5, 9, 18]. Dried parsley has a particularly high level of apigenin that far exceeds any other vegetables or herbs [5]. Chamomile tea, high in apigenin content, is one of the most common sources of apigenin intake from a single ingredient [19]. In nature, apigenin is typically found in a glycosylated form, with the tricyclic core structure linked to a sugar moiety through hydroxyl groups (*O*-glycosides) or directly to carbon (*C*-glycosides). The common apigenin glycosides are apiin, apigenin-7-*O*-glucoside, apigenin-8-*C*-glucoside (vitexin), apigenin-6-*C*-glucoside (isovitexin), apigenin-7-*O*-neohesperidoside (rhoifolin), and apigenin-6-*C*-glucoside 8-*C*-arabinoside (schaftoside) [4, 13, 20].

Since apigenin is widely distributed in food items, and a diet high in flavonoids has been reported with many beneficial health effects, an estimation of the daily intake of apigenin could be useful in the correct interpretation of the relationship between health outcomes and apigenin. Though dietary intake values have been obtained for some individual flavonoids [21], when it comes to the flavone, apigenin, relevant research is very limited [22, 23]. Higher estimation of daily dietary intake of flavonoids is 1 g as glycosides or 650 mg as aglycones [24], while another study estimated an average of only 23 mg/day in adults in the Netherlands [12]. In a study around 1990, the mean flavanol and flavone intake among US health professionals was approximately 20-22 mg per day [25]. In the aforementioned study in the Netherlands in 1988, most of the average daily intake was attributed to quercetin, about 16 mg/day, and the actual daily intake of apigenin was only about 0.69 mg/day [12]. However, another study on the Dutch diet estimated that the average daily intake of apigenin is about 1 mg [22]. A similar number, 1.5 ± 4.9 mg/day (range 0-30.3), was reported in a group of female Flemish dietitians [23]. A more recent paper provided information on the mean intake of flavonoid compounds in adults in the European Union by country, region, and overall [26]. The European average of apigenin intake is 3 ± 1 mg/day using the food consumption data from the European Food Safety Authority (EFSA) and the FLAVIOLA Food Composition Database. The daily intake level of apigenin is 4.23 mg/day in China [27], while among middle-aged and older women in the US, it is 0.13–1.35 mg/d [28]. This intake value among US women is similar to the reported 0.2-1.3 mg/day among 66,940 married women in a US nurses' health study from 1984 through 2002 [29]. By measuring the intake of apigenin from major dietary sources of flavones in adult Australians, the mean daily intake value was determined to be 0.45 mg/day [30].

The above information indicates that, as a phytochemical present in multiple food sources, quantification of dietary intake for apigenin is difficult and has large variation [21]. Diet varies with geographical location, culture, and specific demographics; and it evolves with time. One primary method for dietary intake level estimation relies on dietary assessment, which includes a diet history interview or food frequency questionnaires, and compares this against a food composition database [21]. The reliability of the information collected from subjects, as well as the inclusiveness, extensiveness [21], and demographic specificity of the database can all impact the accuracy of intake levels obtained. Other than the inaccuracy in the data, more information is available on the daily amount of total or combined groups of flavonoids than a single flavonoid of interest [21].

## 3. Chemical Properties: Solubility and Stability

Understanding of the solubility and stability of apigenin is the prerequisite of experiments that study the antimicrobial effects of apigenin in aqueous solutions. Flavonoids are often found in nature as glycosides and phenolic acids, as esters in the aqueous environment of the plant cell vacuole [7]. Apigenin is the aglycone form and it is present in plants naturally as several apigenin glycosides as mentioned in the previous section. Those glycoside conjugates, primarily as apigenin-7-*O*-glucoside, and acylated derivatives are more water soluble than apigenin [10] and their structures have a major impact on their absorption and bioavailability, with the best bioavailability occurring when apigenin is bound to *β*-glycosides [9].

Apigenin is practically insoluble in highly polar solvents such as water (0.00135 mg/mL), and nonpolar solvents such as silicon fluid (0.0728 mg/mL) and safflower oil (0.0317 mg/mL) [15, 16]. Other reports on apigenin's solubility in aqueous solutions show that it ranges from 0.001 to 1.63 mg/mL in nonpolar solvents [6] and 2.16 *μ*g/mL in phosphate buffer at pH 7.5 [31]. Apigenin is freely soluble in dimethylsulfoxide (DMSO) [11]. One source estimated the solubility to be more than 100 mg/mL [16], while another showed that the approximate solubility of apigenin in ethanol, DMSO, and dimethylformamide (DMF) purged with inert gas to be 0.3, 15, and 25 mg/mL [32]. Flavonoids are also more soluble in methanol than in water [33]. As a result, organic solvents like DMSO [34] and Tween 80 [31] are used to dissolve apigenin prior to their addition to an aqueous solution to increase solubility. Different carriers such as ethosomes [35], polymeric micelles of Pluronic P123 and Solutol HS 15 [36], and carbon nanopowder [37], or self-microemulsifying delivery system [38] are also developed and tested to enhance the efficacy of apigenin. Taking into consideration its high permeability, apigenin is categorized as a Class II drug according to Biopharmaceutics Classification System (BCS), whose characteristics are low solubility and high permeability [31, 39].

Pure apigenin is generally regarded as unstable for long term storage at room temperature, and thus it requires storage at -20°C [9]. For use in experiments, it is recommended that fresh solutions be made as needed [32]. Some researchers have investigated the stability of apigenin under various conditions. After heated reflux in water for 30 min, maceration for 24 hours or microwave irradiation for 5 min under 500 W, 93-95% of apigenin was recovered for each condition [33]. However, ultrasonic extraction caused the product to be degraded with only an 86% recovery rate [33]. Another study showed that, in rat plasma* in vitro*, apigenin was stable under three conditions: 24 hours under room temperature, at least 4 weeks when kept frozen at -20°C, and after at least three freeze-thaw cycles [40, 41].

The decomposition for flavonoids depends on the number of substituents in the flavonoid molecule. Hydroxyl groups promote degradation of flavonoids, whereas sugar moiety and methoxyl groups protect flavonoids from degradation during microwave and ultrasonic-assisted extraction [33]. Therefore, the glucosides, other than having a higher solubility than the aglycone, are also chemically more stable, which may be one of the reasons why they have enhanced biological activities [42].

## 4. Safety: Mutagenicity and Hemolysis Tests

Safety is an important aspect of dietary components. In general, dietary plants containing flavonoids have not been associated with negative health impact, but they are rather considered to be beneficial. Apigenin is known for its low toxicity [9, 11, 43, 44]. Evidence has also shown that a flavonoid-rich diet is inversely associated with cancer risk [45–50]. Therefore, the consumption of flavonoids has been assumed to be safe.

Results of an Ames test, one of the oldest methods employed for* in vitro* testing of carcinogenicity using* Salmonella* strains [11], showed that apigenin was not mutagenic or toxic when tested alone [51]. Not only was apigenin not mutagenic [52], it protected against multiple genotoxic agents, such as sodium azide, 9-amino acridine [11, 53]. Apigenin prevented the reverted mutations as a result of sodium azide exposure using histidine auxotroph* S. typhimurium* TA100 strain and the hindrance percent of apigenin was 98.17% [54]. Apigenin-7-*O*-glucoside inhibited sodium azide mutagenicity in* S. typhimurium* TA1535 at 6.67 nM in the top agar when using the layered agar method with an inhibition rate of 27.2% and 9-aminoacridine in* S. typhimurium* TA1537 at 3.33 nM with an inhibition rate of 91.1%. In the yeast deletion assay using mutagens ethyl methanesulfonate and acridine, the inhibition rates were from 4% to 57.7% [53].

Apart from mutagenesis, hemolytic activity is another measurement of safety. Hemolytic tests showed that the percent hemolysis of apigenin was below the permissible limit of 5% after 30 min treatment. Being hemocompatible indicates that apigenin is safe for intravenous application, and suggests that apigenin is non-toxic in mammalian systems [55]. Apigenin was able to significantly attenuate the hemolytic activity of the purified Pneumolysin, the pore-forming toxin secreted by* S. pneumoniae*, in a concentration-dependent manner [56].

As can be concluded from the Ames tests and hemolytic tests, apigenin is not mutagenic nor hemolytic. Taking apigenin glucosides through a daily diet can hardly reach the therapeutic doses used in clinical trial [4] and has not been reported to be harmful. However, as consumers intentionally increase apigenin intake from dietary supplements or pharmaceutical sources, safety concerns may arise with a higher level of apigenin exposure.

## 5. Pharmacokinetics: Absorption, Distribution, Metabolism, and Excretion

The relevance of pharmacokinetics of apigenin in this review is that it determines how much apigenin consumed orally is available to the human gut microbiota. A summary of ADME and drug interactions of apigenin can be found in the review by Tang et al. [4]. In this section, information is provided to show that oral bioavailability of apigenin is poor; it is either excreted unabsorbed in the urine or feces or rapidly metabolized after absorption [4].

### 5.1. Absorption

About 5-10% of total polyphenol intake, mostly monomers and dimers, may be absorbed in the small intestine [17]. The gastrointestinal tract plays a significant role in the metabolism and conjugation of apigenin before the entry of apigenin into the systemic circulation and the liver [57]. In a perfused rat intestinal model, aglycone apigenin was rapidly absorbed [57]. Apigenin can be transported by both passive and active carrier-mediated saturable mechanism in the duodenum and jejunum, and by passive transport mechanisms in the ileum and colon, primarily [31]. Oral administration of apigenin resulted in a peak concentration (C_max_) 1.33 ± 0.24 *μ*g/mL and area under the curve (AUC_0–t_) of 11.76 ± 1.52 *μ*g · hour/mL in rats, which were very low blood apigenin levels [58]. Conflicting conclusions have been drawn regarding the rate of absorption of apigenin. One study on rats concluded that apigenin has a slow absorption because after a single oral administration of radiolabeled apigenin, radioactivity appeared in the blood 24 hours later [59]. While another study reached the opposite conclusion that apigenin had a quick absorption rate in rats as the plasma concentration of apigenin reached the peak level 3.9 hours after oral intake of apigenin in the form of* Chrysanthemum morifolium* extract [60].

### 5.2. Distribution

Multiple studies have been performed to measure the distribution and excretion of apigenin* in vivo*. Following an IV bolus injection of apigenin at 20 mg/kg, the mean value of systemic clearance was 6.12 ± 0.79 L/h/kg [61]. In the previously mentioned study with rats, after a single oral administration of radiolabeled apigenin, the elimination half-time was high with a value of 91.8 hours [59]. The distribution volume was 259 mL, and the plasmatic clearance was 1.95 mL/h [59]. After 10 days, 1.2% of the radioactivity was recovered in the blood, 0.4% in the kidneys, 9.4% in the intestine, 1.2% in the liver, and 24.8% in the rest of the body [59]. About half the apigenin consumed went into urine and feces [59]. In another study, mice were fed a diet containing apigenin for 5, 6, or 7 days. It was found that with a dose of 1.1 mmol/kg apigenin, plasma levels of apigenin reached steady state after 5 days and the steady-state concentrations of apigenin in plasma, liver, and the small intestinal mucosa were 0.09 ± 0.08 nmol/mL, 1.5 ± 1.0 nmol/g, and 86 ± 47 nmol/g, respectively [62]. This is in the similar range to the other study. Apigenin distributes well into the tissues [57].

### 5.3. Metabolism

The absorbed apigenin may go through extensive Phase I and Phase II metabolism [17]. In the rat liver, metabolism of apigenin was found to involve Phase I Enzymes in the presence of NADPH (nicotinamide adenine dinucleotide phosphate), P450 (cytochrome P450 enzymes), or FMO (flavin-containing monooxygenase) [4]. Phase II biotransformation of apigenin involves both enteric and enterohepatic cycling [60]. The conjugation reactions glucuronidation and sulfation are the essential phase II metabolic pathways of apigenin [4]. In both rats and humans, apigenin has been reported to produce major metabolites of glucuronidated and sulfated conjugates [63]. The major hepatic metabolite of apigenin is luteolin [59]. The absorbed apigenin in blood circulation and tissues is in the form of glucuronide, sulfate conjugates, or luteolin [4]. There were three *β*-monoglucuronides that appeared after a glucuronidation reaction, while only one product appeared after a sulfation reaction [4]. In human hepatic cell line, Hep G2, as well as intestinal cell line, Caco-2, apigenin-induced phase II detoxifying enzyme UDP-glucuronosyltransferase UGT1A1 [64, 65]. In addition, glucuronidation reactions also occur in the intestine, and intestinal disposition may be more important than hepatic disposition in the first-pass metabolism of apigenin [63].

### 5.4. Excretion

Excretion of apigenin after oral intake through feces is a good indication of the phenomenon that dietary apigenin is available for metabolism by the gut microbiota. In one of the aforementioned experiments in Section 5.1 using rats, after a single oral administration of radiolabeled apigenin, 51.0% of the radioactivity was recovered in urine and 12.0% in feces within 10 days. In the same research, it was discovered that sex and age of the rats affected apigenin conjugates eliminated via the urinary route [59]. That research also concluded that apigenin has a slow metabolism and a slow elimination phase. Thus, this flavonoid possibly accumulates in the body [59].

### 5.5. Apigenin Glycosides

It is worth mentioning again that apigenin exists in plants as glycosides naturally; therefore, the absorption of the apigenin glycosides and the bioavailability after consumption of glycosides are useful information. Nevertheless, such information on apigenin glycosides is rare, and most research done looked into other flavonoids, in particular quercetin [66, 67]. In fact, in comparison to the other classes of flavonoids, little is known about the bioavailability of flavones [68].

Limited information on the absorption and distribution of apigenin glycosides can only be inferred from a couple of studies available. In the first one, purified flavonoid extract from parsley comprised a mixture of glycosides of the flavones: apigenin, luteolin and chrysoeriol, and of the flavonols: kaempferol, quercetin, and isorhamnetin were administrated to rats by gavage [37]. The dose corresponded to 6.9 mg aglycones per kg body mass. Glycosides were detected by HPLC in the wall and the lumen of the stomach, the wall of the small intestine after 1 hour and 4 hours as well as the cecum wall after 4 hours; however, no concentration values were provided and the glycosides were a mixture of different flavonoids. The apigenin aglycone was detected in the lumen of the small intestine after 1 and 4 hours at the concentrations of 855.59 *μ*g/gram dry matter and 207.05 *μ*g/gram dry matter, respectively; the concentration in cecum after 4 hours was 353.38 *μ*g/gram dry matter. The concentrations of apigenin aglycone in the wall of the stomach, the small intestine, and the cecum were much lower, 16.27 *μ*g/gram dry matter in the stomach wall after 4 hours and 12.58 *μ*g/gram dry matter in the cecum wall. No apigenin was detected in the small intestine wall at either time points. As the treatment comprised of a mixture of flavonoids, the exact source of the apigenin detected was not able to be determined. Nevertheless, as can be seen from the results, the glycosides transported to the cecum were mostly absorbed or deglycosylated rapidly as the cecum luminal contents flavonoids contained only aglycones. Therefore, when studying the impact of dietary apigenin in the colon by* in vitro* simulation, such as when using bioreactors to culture gut microbiota, the treatment group should use apigenin, the aglycone form, rather than naturally present glycosides. In another study, three groups of rats received Apigenin-7-*O*-*β*-D-glucoside solution via tail vein injection at a single dose of 27.0, 18.0, or 9.0 mg/kg [69]. Plasma level of Apigenin-7-*O*-*β*-D-glucoside was measured over the course of 5 hours after drug administration. Estimated plasma concentrations of Apigenin-7-*O*-*β*-D-glucoside based on the figure were about 1, 0.65, and 0.5 *μ*g/gram right after the injection, and decreased to about 0.3, 0.25, and 0.38 *μ*g/gram after one hour for each group.

Depending on the sugar moiety, the absorption could take place in the small intestine or in the colon after deglycosidation [67]. With some exceptions, glucosides are generally the only glycosides that can be absorbed from the small intestine [66]. The absorption of flavonoid glycosides from the small intestine involves the glucose transport pathway [70]. Epithelial *β*-glucosidase-mediated deglycosylation is a critical step in the absorption and metabolism of dietary flavonoid glycosides. It is hypothesized that apigenin glucosides can be hydrolyzed into apigenin by cytosolic *β*-glucosidase (CBG) and lactase-phlorizin hydrolase (LPH), which are enzymes produced by the liver or intestinal cells, or the gut microbiota [4, 71, 72]. LPH has been shown to hydrolyze flavonoid glycosides and is proposed to be brush-border enzymes [57], and the resulting aglycone may then enter epithelial cells by passive diffusion [68]. CBG functions inside cells [71]. The sugar transporter SGLT1 may facilitate the absorption of certain glycosides into epithelial cells [68]; however, apigenin and its various forms of glycosides (apigenin-6-*C*-glucoside, apigenin-7-*O*-glucoside, apigenin-8-*O*-glucoside) did not seem to be absorbed in intact form via the human sodium-coupled glucose transporter hSGLT1 [73].

Flavonoids that cannot be absorbed from the small intestine, as well as the absorbed flavonoids secreted with bile, will be degraded in the colon by the microbiota [66].

In one study, 28.6% and 16.6% of apigenin were recovered in feces and in urine after feeding* Chrysanthemum morifolium* extract to rats [60]. The cumulative apigenin excreted in the bile was 6.34% of the dose [60]. The accumulation of apigenin was also proposed in this study because of the quick absorption rate and the slow elimination phase identified [60].

In one randomized cross-over human intervention study, a basic diet was supplemented with chopped parsley, providing 3.73-4.49 mg apigenin/ Megajoule (approximately 51 mg of apigenin equivalent each day, mainly as apigenin-7-apioside (apiin)) for one week, and the excretion of apigenin was measured [74]. The average urinary excretion of apigenin was significantly higher during intervention with parsley (20.7-5727.3 *μ*g/24 hours) than during the basic diet (0-1571.7 *μ*g/24 hours). No difference between males and females was observed in the mean excretion of apigenin. The amount of apigenin excreted ended abruptly after parsley supplementation was halted. Therefore, urinary excretion and clearance of apigenin are quick, with the excretion half-life estimated to be approximately 12 hours. One missed opportunity in the study was that the plasma concentrations were not measured.

## 6. Usage as Herbal Medicine or Functional Food

Pharmacological potential of apigenin may be reflected by the use of plants containing it as herbal medicine or functional food in different cultures. Plants containing apigenin, along with other flavonoids, have been used to battle diseases in many cultures. Apigenin has been identified as an active ingredient in* Scutellaria barbata* D. Don (Lamiaceae) [75],* Castanea sativa* Mill. (Fagaceae) [76],* Portulaca oleracea* L. [77],* Marrubium globosum* ssp.* Libanoticum* [78],* Combretum erythrophyllum* (Combretaceae) [79],* Aquilegia oxysepala* [80], and propolis [81], among which most are traditional herbal or alternative medicines. Chamomile tea, which is extremely rich in apigenin, has been used as a folk medicine for relieving indigestion or gastritis [9]. Chamomile is also used in mouth rinse, skin care products, and vapor inhalant to reduce inflammation [9]. There are two types of chamomile: the German chamomile and the Roman chamomile, with the former more common as a dietary supplement [26]. Some preliminary studies suggest that a chamomile dietary supplement might be helpful for generalized anxiety disorder (GAD). Researchers conducted a randomized, double-blind, placebo-controlled trial to test the effects of chamomile extract in patients diagnosed with mild to moderate GAD. Compared with placebo, chamomile was associated with a clinically meaningful and statistically significant greater reduction in mean Hamilton Anxiety Rating (HAM-A) scores [82]. Although the researchers suggest that other chamomile species, preparations, and formulations might produce different results [82]. It has been shown that apigenin and other constituents of chamomile bind to BZ receptors and reduce GABA-activated activity and may produce anxiolytic activity [82].

As for the safety of chamomile, there have been reports of allergic reactions as well as interactions between chamomile and cyclosporine and warfarin, which can cause serious consequences [26].

## 7. Pharmacological Activities of Apigenin

As one of the five major flavonoids in plants, apigenin has been extensively studied for its biological functions. Several reviews are available on the bioactivities of apigenin focusing on different aspects including its health functionality [11] and cancer chemoprevention potential [9, 10, 13]. These reviews captured the large body of research on pharmacological activities of apigenin and provided expert opinions on this subject.

Research on apigenin first began in the 1960s, and it was proposed to be chemo-preventative in the 1980s [9, 13, 51]. Recently, apigenin has received much attention because it has low intrinsic toxicity [4, 44] and it exerts differential effects on normal versus cancer cell growth, survival, or apoptosis in several different types of cells [9, 13, 52].

The reported biological functions of apigenin include anti-oxidant, anti-mutagenic, anti-carcinogenic, anti-inflammatory, anti-proliferative, and anti-progression [9]. Apigenin is a moderate anti-oxidant compound because the double bond at the 2,3 carbon makes the structure more reactive, despite the absence of the hydroxyl group at position 3 and a catechol structure in the B-ring [83, 84]. Apigenin is a very effective anti-inflammatory agent compared to other flavonoids. Apigenin was the most potent inhibitor of transcriptional activation of both inducible cyclooxygenase (COX-2) and inducible nitric oxide synthase (iNOS) in lipopolysaccharide (LPS)-activated RAW 264.7 cells among flavonoids such as wogonin, luteolin, tectorigenin, kaempferol, and quercetin, etc., reducing the production of nitric oxide [85, 86]. Apigenin also suppressed nitric oxide production in LPS/gamma-interferon (IFN-*γ*) stimulated C6 astrocyte cells in a dose-dependent manner with an IC_50_ less than 10^−3^M [87]. When tested to see if flavonoids could inhibit LPS-induced TNF-*α* secretion in primary bone marrow-derived mouse macrophages, apigenin was not as effective as quercetin, luteolin, or genistein, but was similar to kaempferol, diosmetin, and hesperetin. It seems that the double bond at C_2_-C_3_ and the position of the B-ring at 2 contribute to the high anti-inflammatory effect [88]. Apigenin inhibits the production of proinflammatory cytokines IL-1*β*, IL-8, and TNF in lipopolysaccharide-stimulated human monocytes and mouse macrophages* in vitro* (concentrations 0.1-25 *μ*M or 0.027-6.756 *μ*g/mL) [89]. It also inhibited TNF-induced NF-*κ*B transcriptional activation in NIH/3T3 cells (concentrations 10-30 *μ*M or 2.702-8.107 *μ*g/mL) [90]. Carrageenan can induce acute paw edema in mice, and the inflammation could be alleviated by apigenin [90]. Apigenin exhibited an anti-inflammatory effect on murine microglia cell line by reducing the production of nitric oxide and prostaglandin E_2_ and was found to be protective against ischemia in neuronal cells (concentrations 1-10 *μ*M or 0.270-2.702 *μ*g/mL) [91]. Apigenin exhibited the highest DNA protective effects against free radicals generated by Fe^2+^ among luteolin and quercetin at the concentration of 1 *μ*M* in vitro*, which indicated its function as an antioxidant (concentration at 10 *μ*M or 2.702 *μ*g/mL) [92]. The anti-inflammatory activity of apigenin has also been reported in lipopolysaccharide-induced inflammation in acute lung injury* in situ* (concentrations 10 and 20 mg/kg body weight) [93].

The anti-mutagenic effect has been reported in* in vitro* cell models,* in vivo* experiments, and AMES test using bacterial models showing that apigenin could prevent, inhibit, or reverse chemically induced genotoxicity [9, 94–96]. The anti-carcinogenic effect of apigenin has been widely reported. Apigenin has been found to be protective against multiple types of cancer including breast cancer [18], cervical cancer, colon cancer, leukemia (concentrations 0-200 *μ*M or 0.0-54.048 *μ*g/mL) [97], lung cancer, prostate cancer (concentrations 0.0-80 *μ*M or 0.0-21.619 *μ*g/mL) [98], skin cancer, thyroid cancer, endometrial cancer, neuroblastoma, and adrenocortical cancer [9, 10]. Derivatized compounds based on apigenin displayed higher antiproliferative activity in human lung, cervical, hepatocellular liver and breast cancer cell lines than apigenin itself (concentrations 62.5-2000 *μ*g/mL) [99].

Concentrations used in research on the pharmacological activities of apigenin should be based on the concentrations systemically reached following dietary intake of the flavonoid. While researchers can use concentrations much higher in the experiments, results obtained from the higher concentrations may not accurately describe the effects in real life when the source of apigenin is from the diet. Information on the distribution of apigenin and hence concentration in different parts of the body after oral consumption of apigenin in human is very rare. Based on available information that the dietary intake of apigenin ranged from 0-4.23 mg/Day, the concentrations used in the above-mentioned researches tend to be higher than what can be realistically achieved after apigenin intake from diet.

Apigenin showed synergistic effects with antitumor drugs such as paclitaxel, 5-fluorouracil, PLX4032 and N-(4-hydroxyphenyl) retinamide by enhancing their bioavailability or efficacy [4]. Apigenin may also serve as a dietary supplement along with small molecule inhibitors to improve radioiodine therapeutic efficacy on invasive tumor margins and thus minimizing future metastasis [15].

## 8. Antimicrobial Effects of Apigenin and Mechanism

The antimicrobial effects of dietary flavonoids have been studied extensively. Although there are many publications reporting the findings on apigenin, there is no review that summarizes the findings. The following section aims to include available information in publications and to make meaningful conclusions.

### 8.1. Antibacterial Activities

The antibacterial potential of apigenin has been tested against many bacteria species and various strains within the same species. Broth microdilution and agar dilution methods are the most popular methods in which the minimal inhibitory concentrations (MICs) are determined as the lowest concentration of treatment that showed no growth after incubation [100, 103, 105, 108]. However, results obtained from the two methods may not necessarily coincide [106]. It has been suggested this might result from the different solubilities of the tested compounds in liquid and the agar gel culture media, and the variability in MIC judgment criteria [110]. Results of the antibacterial and antifungal tests of apigenin measured by its MICs are summarized in Table 1. Due to the limited amount of apigenin that could dissolve in the media, apigenin is considered not active if the MIC is above 128 *μ*g/mL in this review. MIC values are strain-specific, making it difficult to summarize the effects of apigenin in a shorter and more general way.

Apigenin could not inhibit the growth of* Staphylococcus aureus* (8325-4, ATCC 29213, wood 46, and BAA-1717) [108]. Despite the reported lack of activity against* S. aureus*, apigenin was found to remarkably decrease the level of *α*-hemolysin at low concentrations in a concentration-dependent manner in* S. aureus* culture supernatants [108]. Alpha-hemolysin is a pore-forming cytotoxin that is secreted by most* S. aureus *strains, essential for the pathogenesis of* S. aureus* pneumonia [108]. Apigenin protected the adenocarcinomic human alveolar basal epithelial cells (A549 cells) from *α*-hemolysin-mediated injury in the A549 cells and* S. aureus* co-culture system [108]. Therefore, the protective effect apigenin did not come from a reduction in bacterial quantity, but more likely the altered cell physiology. What is more promising is that apigenin alleviated injury of the lung tissue and decreased cytokine levels in the bronchoalveolar lavage fluid in the mouse model of* S. aureus *pneumonia [108]. When apigenin was applied with LysGH15, the lysin derived from phage GH15 with high efficiency and a broad lytic spectrum against MRSA, synergism was observed using a mouse* S. aureus* pneumonia model [111].

Reverse antibiotics (RA) are chemicals that are ineffective against antibiotic-susceptible bacteria but active against the relevant antibiotic-resistant bacteria [112]. RAs can help put a stop to the accumulation of antibiotic resistance by bacteria, because treatment with RA kills the bacteria that have acquired the antibiotic resistance and leaves only the bacteria that possess the original phenotype resistant to RA and susceptible to that antibiotic [112]. Apigenin has RA activities against quinolone-resistant* S. aureus* [107]. The minimum inhibitory concentrations (MICs) of apigenin against quinolone-resistant* S. aureus* strain Mu50 and quinolone-susceptible* S. aureus* FDA 209P are 4 mg/L and more than 128 mg/L [107]. Apigenin was also found to reverse bacterial resistance to cephalosporin ceftazidime in* Enterobacter cloacae *[102]. The MIC of apigenin in ceftazidime-resistant* E. cloacae* (CREC) was higher than 512 *μ*g/mL, which indicated no inhibitive effects [102]. Ceftazidime applied in combination with apigenin showed a synergistic effect with a fractional inhibitory concentration index smaller than 0.01 [102]. The 5,7- OH group of A ring and one 4′- OH group of the B ring in apigenin were found important in reversing antimicrobial resistance [102]. The significantly enhanced activities of ceftazidime by apigenin may have been the result of peptidoglycan synthesis inhibition, certain *β*-lactamase enzymes inhibition, and alteration of outer membrane and cytoplasmic membrane permeabilization [102].

Contrasting results have been observed on apigenin's effect on* Helicobacter pylori in vitro*. While one study saw no inhibitory effect against thirteen randomly selected clinical strains of* H. pylori* from antral biopsies and a reference strain* H. pylori* ATCC 43504 [101], apigenin showed moderate antibacterial activity in another, with a MIC of 25 *μ*g/mL against both* H. pylori* SS1 and* H. pylori* ATCC 43504 [106]. An* in vivo* experiment using Mongolian gerbils, apigenin treatments (30–60 mg/kg body weight/day) effectively decreased* H. pylori*-induced atrophic gastritis and* N*′-methyl-*N*'-nitro-*N*-nitroso-guanidine (MNNG)-induced dysplasia/gastric cancer rates [113]. The dose of 60 mg apigenin/kg bodyweight/day significantly decreased* H. pylori* colonization and* H. pylori*-induced histological changes of neutrophil and monocyte infiltrations and atrophic gastritis [113].

*Streptococcus mutans* is the main pathogen responsible for the development of dental caries in humans. The organism synthesizes glucans during adhesive interactions with the tooth surface and other oral bacteria. Apigenin was found capable of inhibiting water-insoluble glucans synthesis and reducing the incidence of dental caries with minimal effects on the viability of oral microbiota populations* in vivo* when applied topically [114, 115].

The antibacterial activity of apigenin-*C*-8 glucoside was reported to be weak compared to the apigenin aglycone [116]. It was hypothesized that glycosylation causes a reduction in lipophilicity and consequently diminishes the ability to penetrate bacterial membrane [116]. Some derivatives of apigenin showed increased antibacterial activity against* S. aureus*,* Bacillus subtilis*,* Escherichia coli*, and* Pseudomonas aeruginosa*, especially 7-[3-(Morpholin-4-yl)propoxy]-5-hydroxy-2-(4-hydroxyphenyl)-4H-chromen-4-one [99]. Given the prevalence of antibiotic resistance, apigenin or its derivatives could be a candidate as a new antibiotic or as a dietary supplement to enhance the performance of antibiotics.

There are many other studies that tested apigenin-containing plant extracts' antimicrobial effects. Plant extracts could have several other bioactive components, thus further research is needed to accurately attribute the cause of antimicrobial effect. Those studies that did not test the effects of apigenin independent of other potential bioactive compounds are not included in this review.

Some insight is available on the mechanism of the antibacterial activity of apigenin. It has been previously indicated that the main targets of apigenin on bacteria may be the nucleic acid processing enzymes and cell wall/membrane [34, 106]. In a study where apigenin inhibited* S. aureus*, mode of action was compared to other antibiotics with known mechanism by clustering the treatments based on intracellular metabolites. Apigenin was clustered with rifampicin and norfloxacin which target RNA polymerase, and gyrase and topoisomerase IV, respectively [80], which indicates that the target of apigenin could be nucleic acid processing enzymes [75]. The target DNA gyrase was also reported in one other study [117]. Apigenin affects the d-Alanine: d-Alanine ligase and the type II fatty acid synthetic pathway, both of which are involved in cell membrane/wall synthesis [34]. In apigenin-treated* E. caccae*, the expression of stress response genes and protein chaperone genes were found to be up-regulated, which indicated the overall adverse effects of apigenin on the bacterium [34].

Although the methods employed in determining the inhibitory effects are different, nearly all studies focused on pathogenic bacteria and cultured the bacteria under aerobic conditions. Since dietary apigenin will enter the colon, it is worthwhile to study the effects of apigenin on commensal gut bacteria under anaerobic culture conditions.

### 8.2. Antiviral Activity

Apigenin has been reported to be able to inhibit multiple viruses, including enterovirus 71 (EV71), herpes simplex virus HSV-1 and HSV-2, hepatitis C virus, influenza virus, hand, foot, and mouth disease virus, and African swine fever virus (ASFV), but not coxsackievirus A16 (CAV16). The details are described as follows.

One of the major causative agents for hand, foot, and mouth disease (HFMD), EV71, is a member of genus* Enterovirus* in Picornaviridae family [118]. Research found that apigenin inhibited EV71-mediated cytopathogenic effect and EV71 replication* in vitro* [118]. Viral polyprotein expression, EV71-induced cell apoptosis, intracellular reactive oxygen species (ROS) generation and cytokines up-regulation were inhibited [118]. Apigenin could interfere with viral internal ribosome entry site (IRES) activity and inhibit EV71-induced c-Jun N-terminal kinase (JNK) activation which is critical for viral replication [118]. Another study tested methanol extract of* Paulownia tomentosa* flower for antiviral activity against enterovirus 71 (EV71) and CAV16* in vitro* [119]. CAV16 is another one of the predominant etiologic agents of hand, foot, and mouth disease [119]. The extract had no effect against CAV16 infection; however, apigenin was again identified as an active component to inhibit EV71 [119]. The EC_50_ value, inhibitory concentration of compound that produces 50% inhibition of virus-induced cytopathic effects, for apigenin to block EV71 infection was 11.0 *μ*M, with a selectivity index (SI) of approximately 9.3 [119]. Similar compounds like naringenin and quercetin were not active against EV71 infection [119].

In another study, apigenin, as a bioactive compound in* Ocimum basilicum*, also known as sweet basil, showed a broad spectrum of antiviral activity [120]. The highest activities were against Herpes simplex virus HSV-2 (EC_50_ = 9.7 mg/L; SI = 6.2), adenovirus ADV-3 (EC_50_ = 11.1 mg/L; selectivity index SI = 5.4), hepatitis B surface antigen (EC_50_ = 7.1 mg/L; SI = 2.3) and hepatitis B e antigen (EC_50_ = 12.8 mg/L; SI = 1.3). Apigenin inhibited HSV-1 in Madin-Darby canine kidney (MDBK) cells within the concentrations of 0.4–1.6 *μ*g/ml [100].

Apigenin was found to be able to inhibit Hepatitis C virus (HCV) replication* in vitro *[121]. Apigenin decreased the expression levels of mature microRNA miR122, a liver-specific miRNA that positively regulates HCV replication, through inhibition of TRBP (transactivating response RNA-binding protein) phosphorylation without significantly affecting cell growth [121]. Therefore, apigenin intake, either through regular diet or supplements, may be beneficial for chronically infected patients [121].

Apigenin showed significant anti-influenza virus activities with IC_50_ of 1.34 *μ*g/mL in MDBK cells [122].

Animal disease related viruses were also found to be inhibited by apigenin. Foot-and-mouth disease (FMD) is a highly contagious and clinically acute viral disease of domestic and wild cloven-hoofed animals worldwide caused by FMD virus (FMDV) [123]. FMDV belongs to the* Aphthovirus* genus of the Picornaviridae family [123].* In vitro* experiment shows that apigenin inhibited FMDV infection at the viral post-entry stage with no extracellular virucidal activity [123]. Similar to EV71, apigenin interfered with the translational activity of FMDV by internal ribosome entry site [123].

ASFV causes serious diseases in domestic pigs. A dose-dependent anti-ASFV effect of apigenin was reported* in vitro* [124]. Apigenin was highly effective at the early stages of infection and was able to achieve a more than 3-log reduction in ASFV yield when it was added to Vero cells at 1-hour post-infection [124]. Apigenin inhibited ASFV-specific protein synthesis and viral factory formation. Continuous apigenin treatment prevented cytopathic effect in ASFV-infected cells [124].

### 8.3. Antifungal Activity

*Candida albicans* ATCC 10231 and* C. parapsilosis* ATCC 22019 were reported to be inhibited by apigenin with MIC of 8 and 16 *μ*g/mL, respectively [100]. Apigenin could be used as an antifungal agent in the clinical treatment of dermatophytosis [125]. Mice were experimentally induced to develop dermatophytosis with* Trichophyton mentagrophytes* and lesions were treated with two concentrations of apigenin ointment, 2.5 mg/g, and 5mg/g. Apigenin exhibited a similar effect as the reference drug Terbinafine [125]. Applied at 5 mg/g for both apigenin and Terbinafine, complete recovery from the infection was recorded on the 12th day in both groups. With 2.5 mg/g ointment, the infection was cured on the 16th day [125]. A recent paper reported that apigenin induced cell shrinkage in* C. albicans*, altering the cell membrane potential and causing leakage of intracellular components [126].

### 8.4. Antiparasitic Activity

Leishmaniasis is a disease that affects more than 12 million people worldwide with around 2 million new cases each year. It is caused by the protozoa parasite* L. amazonesis *[127]. Apigenin treatment for 24 hours resulted in concentration-dependent inhibition of cellular proliferation with IC_50_ equals 23.7 *μ*M and increased reactive oxygen species (ROS) generation. Other mechanisms of the negative effects of apigenin on* L. amazonesis* include extensive swelling in parasite mitochondria, altered mitochondrial membrane potential, rupture of the trans-Golgi network, and cytoplasmic vacuolization [128].

## 9. Apigenin and Human Gut Microbiota

As shown earlier in the ADME section of the review, part of the apigenin intake is excreted in the feces and apigenin aglycone was detected in cecum luminal content, thus the bacterial community in the colon is exposed to dietary apigenin. Once apigenin enters the colon, it becomes the substrate of the pool of various enzymes produced by the gut microbiota. Human gut microbiota has been found to harbor enzymes that could degrade apigenin.

Some, not all, commensal gut bacteria are capable of degrading apigenin on their own.* Bacteroides distasonis* was found to be capable of converting apigenin-7-*O*-glucoside to apigenin; however, it was not the case with* E. coli *[129].* Eubacterium ramulus*, a strictly anaerobic human gut bacterium, is able to metabolize apigenin, as well as quercetin, naringenin, daidzein and genistein [130]. It possesses a phloretin-hydrolase able to break the phloretine C-C bond [131]. Another anaerobic bacterium isolated from human feces,* Clostridium orbiscindens*, was found to degrade apigenin to 3-(4-hydroxyphenyl)propionic acid with phloretin and naringenin as the two intermediates [132]. This degradation product 3-(4-hydroxyphenyl)propionic acid, also known as desaminotyrosine (DAT) is beneficial during influenza by triggering type I interferon signaling and in turn augmenting antiviral responses by phagocytic cells; therefore preventing inflammation and severe disease [133]. It is possible that the anti-inflammatory activity and protective effects on lung tissues mentioned in the previous sections are mediated by microbial metabolites from apigenin as well [93, 108]. Degradation of apigenin and its glycosides most likely involves multiple bacteria, with complementary and overlapping functionalities.

Some information is available on how the human gut microbiota metabolizes apigenin glycosides using* in vitro *experiments or animal models. Hanske et al. performed a study demonstrating that the bioavailability of apigenin-7-*O*-glucoside is influenced by human intestinal microbiota using rats and* in vitro* culture of human gut microbiota in test tubes [129]. The* in vitro* culture experiment was carried out by incubating 10 mL culture of human gut microbiota and apigenin-7-*O*-glucoside mixture in airtight tubes for 24 hours at 37°C [129]. Results showed that human fecal suspensions converted apigenin-7-*O*-glucoside completely within 5 hours of incubation [129]. Apigenin-7-*O*-glucoside concentration remained largely stable without the presence of bacteria [129]. Apigenin and naringenin were transiently formed as intermediate metabolites from apigenin-7-*O*-glucoside [129]. The end products of apigenin-7-*O*-glucoside microbial degradation were 3-(4-hydroxyphenyl)propionic acid (4-HPPA) and trace amounts of 3-(3-hydroxyphenyl)propionic acid (3-HPPA) [129]. In rat models, germ-free rats excreted via urine apigenin-7-*O*-glucoside, apigenin, and luteolin uniformly within 48 hours after application of apigenin-7-*O*-glucoside, both in free and conjugated forms [129]. Additional metabolites were excreted by human-microbiota associated (HMA) rats in urine: naringenin, phloretin, 3-(3,4-dihydroxyphenyl)propionic acid (3,4-DHPPA), 4-HPPA, 4-hydroxycinnamic acid (4-HCA), and 3-HPPA, with apigenin-7-*O*-glucoside, apigenin, luteolin, naringenin, phloretin, 4-HPPA, and 4-HCA in both their free and conjugated forms and only free forms of 3,4-DHPPA and 3-HPPA [129]. Apigenin-7-*O*-glucoside, apigenin, and luteolin, mainly as conjugates, were observed in germ-free rats' fecal excretion, while they were excreted at a considerably lower level in HMA rats [129]. This study strongly supports that the gut microbiota plays a major role in the metabolism of dietary apigenin.

Another study using an* in vitro* system to study the fermentation of apigenin showed that the number of metabolites formed is donor-dependent with 3-(4-hydroxyphenyl)propionic acid, 3-hydroxyphenyl-acetic acid, and 3-phenylpropionic acid being the common metabolites detected with microbiota samples from all three different donors [134]. This study also found that fermentation rates of* C*-glycosides are slower compared to the rates of* O*-glycosides.

While microbiota facilitates degradation of apigenin, apigenin and its metabolites may also modify the structure and function of gut microbiota considering its effects on bacteria. The effect on the modulation of gut ecology is still poorly understood [17]. Throughout the study by Hanske et al., the similarity of the intestinal microbiota composition of rats to the human fecal sample used to associate the rats was determined by PCR-coupled denaturing gradient gel electrophoresis and was reported to be ranged from 63.3 to 75.8%. However, no detailed information was available on compositional changes of the community although there seemed to be a change. The* in vitro* experiment was carried in test tubes without pH control and a freshly inoculated microbiota. It would be more ideal to perform the* in vitro* incubation of apigenin and gut bacteria in a chemostat and establish a stable community. In the aforementioned research that used samples from three different donors, no significant changes in the microbiota composition and short-chain fatty acid levels as products of carbohydrate fermentation were detected between incubations with different phenolic compounds [134]. In another short-term* in vitro* fermentation experiment that tested the effects of apigenin on the human gut microbiota, apigenin slightly enhanced the overall microbial density and the microbial diversity [34]. Some microbial community composition changes were observed at the phylum level between the apigenin-treated group and the control group [34]. However, those differences may not be big enough to make a practical significance.

## 10. Conclusion

Apigenin has been shown to possess antibacterial, antiviral, antifungal, and antiparasitic activities. The inhibitory effects on bacteria are strain-specific. In some cases, although apigenin was not able to inhibit the growth of pathogenic bacteria, it decreased the production of toxin and alleviated injury caused by the pathogen [108]. Synergy has been observed between apigenin and other antibiotics [105, 110]. Broth microdilution and agar dilution are the most popular methods to determine the minimal inhibitory concentrations; however, due to system differences, there are discrepancies between the values obtained from the two methods. There seems to be no consensus on which method is better in general. The antimicrobial effects of apigenin are strain-specific and limited by its solubility. Still, it is a good starting point to investigate the antimicrobial effects and mechanisms of apigenin, or how to use it to complement or enhance the effectiveness of antibiotics.

Nearly all studies looking at the antibacterial effects of apigenin used pathogenic bacterial and cultured the bacteria under aerobic conditions. Some published research looked at other similar dietary phytochemicals [135–137]. The effect of apigenin on commensal gut bacteria under anaerobic conditions is a new territory to explore.

Initial information on the interactions between apigenin and the human gut microbiota is available. It is now known that a few gut bacteria are able to degrade apigenin into smaller molecules, some of which are better absorbed than apigenin and are more biologically active. Inconclusive results have been reported on how apigenin affects the structure of the gut microbiota; in the real-life scenario, it is most likely that the effects would be mild due to the limited amount of apigenin from dietary sources and the ability of the microbiota to degrade apigenin. We also know that the changes in the microbial community and the rate of degradation of apigenin depend on the form of apigenin, whether it is glycosylated and which type of glycoside, and the gut microbiota composition [134]. Also because of the complexity of human gut microbiota, there is still much to investigate.

Our knowledge of how apigenin affects human gut microbiota and how it in turn modulates human health is still very limited. A powerful tool to study the structural changes in the gut microbiota is Next-Generation Sequencing. High-throughput sequencing platforms have enabled rapid determination of microbiota composition by sequencing and analyzing either targeted regions of certain genes (e.g., 16S gene for bacteria, Internal transcribed spacer gene for fungi) or the whole genome of the community (shot-gun metagenomics). It is yet to be discovered what other gut bacteria can metabolize apigenin and what are the metabolic products. There might be a potential cross-feeding phenomenon in apigenin degradation, as the metabolites from one bacterium could be the substrate for another. The impact of dietary apigenin on human health through gut microbiota can be explored through metabolomics as the smaller molecules produced by the gut microbiota may be more relevant than the structure of the gut microbiota itself. Host characteristics such as gender, age, and disease status may also play a role in affecting the interactions between apigenin and the gut microbiota.

## Figures and Tables

**Table 1:** The antibacterial and antifungal effects of apigenin measured by its minimum inhibitory concentration (MIC). ATCC, American type culture collection; RSKK, Refik Saydam Central Hygiene Institute. MRSA, methicillin-resistant *Staphylococcus aureus*; MSSA, methicillin-susceptible *Staphylococcus aureus*; ES*β*L+, extended-spectrum beta-lactamases; NCTC, National Collection of Type Cultures; NCIMB, National Collection of Industrial, Food and Marine Bacteria; MYA, Meyer et Yarrow; OD, Optical Density.

Species	Strain information	MIC *μ*g/mL	Method of determining MIC	Reference
Antibacterial activity				

*Acinetobacter baumannii*	RSKK 02026	2	Broth microdilution with no growth under the microscope	[100]
	clinical isolate	64	Broth microdilution with no growth under the microscope	[100]

*Bacillus cereus*	clinical isolate	Not active	Broth microdilution with no growth under the microscope	[101]

*Bacillus subtilis*	ATCC 6633	8	Broth microdilution with no growth under the microscope	[100]
	clinical isolate	16	Broth microdilution with no growth under the microscope	[100]

*Enterobacter aerogenes*	ATCC 13048	64	Broth dilution with no visible growth	[76]

*Enterobacter cloacae*	ATCC 10699	64	Broth dilution with no visible growth	[76]
	ceftazidime-resistant *strain*	> 512	Broth microdilution with no growth	[102]

*Enterococcus faecium*	NCTC 7171	Not active	Agar dilution with strong, moderate, weak, or absent growth	[103]
	NCTC 12202	Not active	Agar dilution with strong, moderate, weak, or absent growth	[103]
	NCTC 12204	Not active	Agar dilution with strong, moderate, weak, or absent growth	[103]

*Enterococcus faecalis*	ATCC 19433	> 400	Broth microdilution with no growth	[104]
	ATCC 29212	8	Broth microdilution with no growth under the microscope	[100]
	NCIMB 775	Not active	Agar dilution with strong, moderate, weak, or absent growth	[103]
	NCTC 12201	Not active	Agar dilution with strong, moderate, weak, or absent growth	[103]
	NCTC 12203	Not active	Agar dilution with strong, moderate, weak, or absent growth	[103]
	clinical isolate	128	Broth microdilution with no growth under the microscope	[100]

*Escherichia coli*	ATCC 25922	135.12	Broth microdilution with no visual turbidity and no growth on agar after incubation	[105]
	ATCC 25922	> 200	Agar dilution with no visible bacterial growth	[106]
	ATCC 11229	128	Broth dilution with no visible growth	[76]
	ATCC 14948	>400	Broth microdilution with no growth	[104]
	ATCC 35218	4	Broth microdilution with no growth under the microscope	[100]
	ATCC 35218	Not active	Broth microdilution with no growth under the microscope	[101]
	JM109	200	Agar dilution with no visible bacterial growth	[106]
	NCTC 11560	Not active	Agar dilution with strong, moderate, weak, or absent growth	[103]
	NCTC 11954	Not active	Agar dilution with strong, moderate, weak, or absent growth	[103]
	NCTC 12241	Not active	Agar dilution with strong, moderate, weak, or absent growth	[103]
	ES*β*L+ clinical isolate	128	Broth microdilution with no growth under the microscope	[100]

*Helicobacter pylori*	ATCC 43504	25	Agar dilution with no visible bacterial growth	[106]
	ATCC 43504	Not active	Broth microdilution with no growth under the microscope	[101]
	SS1	25	Agar dilution with no visible bacterial growth	[106]
	clinical isolates	Not active	Broth microdilution with no growth under the microscope	[101]

*Klebsiella pneumoniae*	ATCC 10031	128	Broth dilution with no visible growth	[76]
	RSKK 574	8	Broth microdilution with no growth under the microscope	[100]
	ES*β*L+ clinical isolate	128	Broth microdilution with no growth under the microscope	[100]

*Mariniluteicoccus flavus*	ATCC 10240	Not active	Broth microdilution with no growth under the microscope	[101]

*Proteus mirabilis*	ATCC 7002	4	Broth microdilution with no growth under the microscope	[100]
	clinical isolate	Not active	Broth microdilution with no growth under the microscope	[101]
	ES*β*L+ clinical isolate	128	Broth microdilution with no growth under the microscope	[100]

*Proteus vulgaris*	ATCC 12454	Not active	Broth dilution with no visible growth	[76]

*Pseudomonas aeruginosa*	NCTC 8203	Not active	Agar dilution with strong, moderate, weak, or absent growth	[103]
	NCTC 8506	Not active	Agar dilution with strong, moderate, weak, or absent growth	[103]
	ATCC 10145	2	Broth microdilution with no growth under the microscope	[100]
	NCTC 10662	Not active	Agar dilution with strong, moderate, weak, or absent growth	[103]
	ATCC 27853	Not active	Broth microdilution with no visual turbidity and no growth on agar after incubation	[105]
	ATCC 27853	Not active	Broth microdilution with no growth under the microscope	[101]
	ATCC 27853	400	Broth microdilution with no growth	[104]
	ATCC 27853	64	Broth dilution with no visible growth	[76]
	clinical isolate	32	Broth microdilution with no growth under the microscope	[100]

*Salmonella enterica*	Serotype Choleraesuis ATCC 10708	>400	Broth microdilution with no growth	[104]

*Staphylococcus aureus*	ATCC 6538	Not active	Broth microdilution with no growth under the microscope	[101]
	FDA 209P (the same as NCTC 7447 or ATCC 6538P)	> 128	Broth microdilution with no visible growth	[107]
	MRSA ATCC 10442	Not active	Broth microdilution with no visual turbidity and no growth on agar after incubation	[105]
	ATCC 10832	> 1024	Broth microdilution with no bacterial growth by OD_600nm_	[108]
	ATCC 13709	no inhibition	Broth dilution with no visible growth	[76]
	ATCC 25923	400	Broth microdilution with no growth	[104]
	ATCC 25923	16	Broth microdilution with no growth under the microscope	[100]
	ATCC 29213	> 1024	Broth microdilution with no bacterial growth by OD_600nm_	[108]
	ATCC 33591	Low activity	Agar dilution with strong, moderate, weak, or absent growth	[103]
	NCIMB 9968	Low activity	Agar dilution with strong, moderate, weak, or absent growth	[103]
	NCTC 6571	Low activity	Agar dilution with strong, moderate, weak, or absent growth	[103]
	NCTC 8325-4	> 1024	Broth microdilution with no bacterial growth by OD_600nm_	[108]
	NCTC 10788	Low activity	Agar dilution with strong, moderate, weak, or absent growth	[103]
	NCTC 11940	Low activity	Agar dilution with strong, moderate, weak, or absent growth	[103]
	NCTC 11561	Low activity	Agar dilution with strong, moderate, weak, or absent growth	[103]
	BAA 1717	> 1024	Broth microdilution with no bacterial growth by OD_600nm_	[108]
	DU 1090	> 1024	Broth microdilution with no bacterial growth by OD_600nm_	[108]
	strain Mu50	4	Broth microdilution with no visible growth	[107]
	20 MRSA and MSSA strains	3.9-15.6	Disc-diffusion test	[75]
	MRSA clinical isolate	> 128	Broth microdilution with no growth under the microscope	[100]
	34 MRSA clinical isolates	> 4000	Agar dilution with no growth	[109]

*Streptococcus pyogenes*	ATCC 12344	135.12	Broth microdilution with no visual turbidity and no growth on agar after incubation	[105]
	ATCC 19615	50	Broth microdilution with no growth	[104]

Antifungal activity				

*Candida albicans*	ATCC 10231	8	Broth microdilution with no growth under the microscope	[100]
	ATCC 90028	Not active	Broth microdilution with no visual turbidity and no growth on agar after incubation	[105]

*Candida glabrata*	ATCC MYA2950	67.56	Broth microdilution with no visual turbidity and no growth on agar after incubation	[105]

*Candida parapsilosis*	ATCC 22019	16	Broth microdilution with no growth under the microscope	[100]
